# A novel radiomics approach for predicting TACE outcomes in hepatocellular carcinoma patients using deep learning for multi-organ segmentation

**DOI:** 10.1038/s41598-024-65630-z

**Published:** 2024-06-26

**Authors:** Krzysztof Bartnik, Mateusz Krzyziński, Tomasz Bartczak, Krzysztof Korzeniowski, Krzysztof Lamparski, Tadeusz Wróblewski, Michał Grąt, Wacław Hołówko, Katarzyna Mech, Joanna Lisowska, Magdalena Januszewicz, Przemysław Biecek

**Affiliations:** 1 Second Department of Radiology, Medical University of Warsaw, Banacha 1a st., 02-097 Warsaw, Poland; 2 Faculty of Mathematics and Information Science, Warsaw University of Technology, Koszykowa 75 st., Warsaw, Poland; 3 Department of General, Transplant and Liver Surgery, Medical University of Warsaw, Banacha 1a st., Warsaw, Poland; 4 Department of General, Gastroenterological and Oncological Surgery, Medical University of Warsaw, Banacha 1a st., Warsaw, Poland

**Keywords:** Outcomes research, Translational research, Computational models, Machine learning

## Abstract

Transarterial chemoembolization (TACE) represent the standard of therapy for non-operative hepatocellular carcinoma (HCC), while prediction of long term treatment outcomes is a complex and multifactorial task. In this study, we present a novel machine learning approach utilizing radiomics features from multiple organ volumes of interest (VOIs) to predict TACE outcomes for 252 HCC patients. Unlike conventional radiomics models requiring laborious manual segmentation limited to tumoral regions, our approach captures information comprehensively across various VOIs using a fully automated, pretrained deep learning model applied to pre-TACE CT images. Evaluation of radiomics random survival forest models against clinical ones using Cox proportional hazard demonstrated comparable performance in predicting overall survival. However, radiomics outperformed clinical models in predicting progression-free survival. Explainable analysis highlighted the significance of non-tumoral VOI features, with their cumulative importance superior to features from the largest liver tumor. The proposed approach overcomes the limitations of manual VOI segmentation, requires no radiologist input and highlight the clinical relevance of features beyond tumor regions. Our findings suggest the potential of this radiomics models in predicting TACE outcomes, with possible implications for other clinical scenarios.

## Introduction

Hepatocellular carcinoma (HCC) presents a challenge given its complex disease course and multilevel treatment strategy^1^. Minimally invasive procedures, such as transarterial chemoembolization (TACE), currently represent the standard of care for patients with non-operative HCC^2^. Most HCC prognostic systems rely on liver function status combined with basic radiologic features^3^. Traditionally, radiologists analyze imaging data using simplifications, concentrating on a few basic imaging features, such as the tumor hyperenhancement or lesion diameter^4^. However, the complexity of medical imaging makes human perception insufficient to fully utilize the potential of diagnostic information^5^. As a result, there is a need for more comprehensive approaches, such as radiomics which rely on the extraction and analysis of quantitative features from medical imaging data.

Overall, outcome prediction following TACE is a challenging and multifactorial task and new risk stratification models are needed. Artificial intelligence (AI) and radiomics has the potential to revolutionize the field of HCC therapy^6,7^. They can be employed to identify “hidden” patterns or to analyze a large number of variables simultaneously^8–11^. For example, recent studies showed that multiphase computed tomography (CT) can be employed to evaluate the progression of liver fibrosis or the severity of chronic liver disease^12–14^. Additionally, conditions such as chronic kidney disease, osteopenia or atherosclerosis, potentially leading to morphological organ changes like kidney shrinkage and of the density of organs, can influence the risk profile of patients with HCC^15–17^. Our approach leverages radiomics features from multiple internal organ VOIs to capture these changes, testing their impact on treatment outcomes. Traditionally, radiomics studies have focused on the laborious manual segmentation of volume of interest (VOI), primarily limited to disease or tumor loci. This approach often overlooks other important VOIs like the spleen or liver parenchyma, which can be crucial, especially in patients with liver cirrhosis. Recently, deep learning models pretrained on large datasets for multiple organ segmentation have been introduced. One of them is “TotalSegmentator”—an open-source model capable of segmenting various anatomical structures from CT images^18^.

In this study, we investigated the potential of a novel CT-based radiomics approach for predicting TACE outcomes in HCC patients. The novelty primarily lies in the adoption of a multi-organ radiomics approach within the context of TACE outcome prediction. This strategy leverages the potential of multiple organ VOIs to comprehensively capture the information encoded in multiphase CT images. We hypothesize that the utilization of such multidimensional data can provide valuable insights into predicting long-term treatment outcomes. This distinguishes our approach from the majority of radiomics studies in oncology that focus mainly on radiomic features derived from tumor regions, often combined with various clinical variables. Moreover, the models are specifically designed for predicting overall survival (OS) and progression-free survival (PFS) with little or no radiologist input. In contrast to other studies, we employed a fully automated organ segmentation process using a pre-trained deep learning model. Finally, we used explainable AI (XAI) techniques to improve the interpretability of the proposed models.

## Materials and methods

### Study design

**Figure 1:**
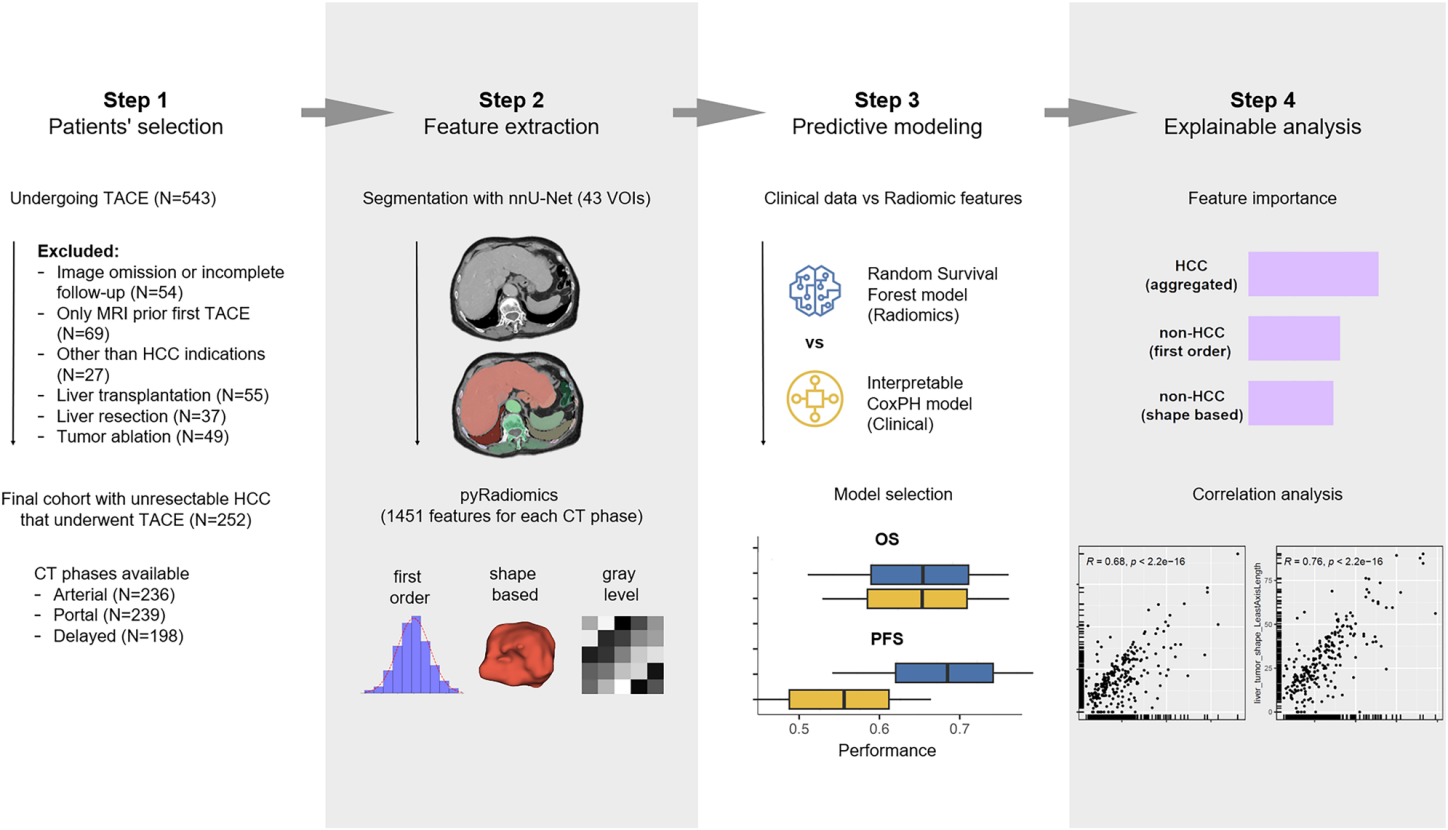
Schematic study flowchart.

In this retrospective, single-center study, we analyzed the data of patients diagnosed with unresectable HCC treated with TACE. The primary study goal was to create radiomics models for the prediction of long-term TACE outcomes, while the outcome measures were OS and PFS. Figure 1 presents a schematic study flowchart. The study was conducted in compliance with the current version of the Declaration of Helsinki. The need for ethical approval and informed consent were waived for this study by the Bioethics Committee at the Medical University of Warsaw due to its retrospective study design.

### Data sources and eligibility criteria

A search of the internal database was conducted for consecutive patients undergoing TACE between 2016 and 2020. A schematic patients’ flowchart is shown in Fig. 1, step 1, while details of eligibility criteria are presented in Supplementary Table S1. The final cohort consisted of N = 252 patients with unresectable HCC who underwent a total of 734 TACE procedures.

#### Pre-TACE assessment

The diagnosis of HCC was made using imaging criteria (LR-5 according to the Liver Imaging Reporting and Data System [LI-RADS])^19^ or pathomorphological examination. All patients underwent a series of laboratory examinations before the initial TACE session. In total, we evaluated 16 clinical variables (Supplementary table S2). Additionally, hepatoma arterial-embolization prognostic (HAP) score^20^, modified hepatoma arterial embolization prognostic II (mHAP-2) score^21^, six and twelve (6&12) score^22^ and ALBI-TAE score^23^ were calculated.

#### Pre-TACE CT imaging

All patients underwent a multiphasic, contrast-enhanced CT within 90 days before the initial TACE cycle (in line with LI-RADS recommendations). Overall, we collected CT examinations of N = 252 patients (including phases: arterial N = 236, portal N = 239, delayed N = 198, details are presented in Supplementary table S3).

#### TACE technique and postprocedural follow-up

All patients underwent standard conventional TACE procedures. Vessels feeding target lesions were selectively catheterized and 20–40 mL of a mixture of lipiodol and doxorubicin in a 1:1 ratio was injected until arterial flow stasis was observed. The TACE procedure was repeated after 4–6 weeks when indicated and feasible. A standard embolization cycle typically consisted of two (or three, if indicated) TACE sessions followed by a subsequent CT or MRI. Favorable treatment response was defined as a LR nonviable category assessed by LI-RADS Treatment Response algorithm (LR-TR). If a LR-TR nonviable response was achieved after the TACE cycle, patients were followed up by serial imaging and serum α-fetoprotein concentration measurements.

The date of the first TACE was adopted as an index day for analysis. The end of the follow-up period was defined as the time of death or the last clinical follow-up (with a minimum duration of 2 years). For patients who achieved a LT-TR nonviable TACE response, PFS was calculated as the interval between achieving a LR-TR nonviable response and the date of reported progression.

### Model deployment

The model creation pipeline involved three main steps: First, a deep learning model was used to automatically generate multiple organ segmentation masks utilized for radiomics feature extraction. Second, machine learning (ML) was employed for the deployment of models aimed at predicting treatment outcomes (OS and PFS). Third, XAI methods were used to enhance the interpretability of the proposed models.

#### Preprocessing of CT images and deep learning VOI segmentation

For each patient, the pre-TACE CT examination was de-identified, converted to NIfTI format and securely stored in a dedicated database. No specific filtering or normalization of CT series were performed in our study. To generate segmentation masks for various anatomical structures, the “TotalSegmentator” model that utilizes the nnU-Net architecture was employed^18,24^. In our study, all imaging phases were segmented separately, each for 102 VOI, while only VOIs of structures that were present in an abdominal CT examination were included for further analyses. Additionally, for segmentation of liver tumors and hepatic vessels, a separate nnU-Net sub-model, the TotalSegmentator extension, was utilized. The full list of N = 43 VOIs included in the final analysis is presented in Supplementary table S4.

#### Feature extraction

Features were extracted using the PyRadiomics framework and categorized into several classes. All features were defined in accordance with the Imaging Biomarker Standardization Initiative. Detailed information about the features and their definitions can be found in a separate document by Zwanenburg et al.^25^. All radiomics features were calculated separately for each whole organ and the largest liver tumor VOIs in each CT phase. For every N = 43 VOI in each CT exam phase, we extracted a set N = 14 shape-based descriptors and N = 18 first-order statistics. Moreover, for the largest liver tumor VOI, N = 75 Gy-level features were extracted. Together, for all VOIs, this gives a total of N = 43 VOIs*32 features + 75 features = 1451 radiomic features for each phase of CT examination (details are presented in Supplementary table S2).

#### Modeling

Two distinct categories of models were developed, namely radiomics and clinical. For the training and subsequent evaluation of each model, a subgroup comprising individuals with complete sets of selected variables was chosen. Moreover, we conducted a comparative analysis with several other state-of-the-art prognostic models deployed on our dataset, including ALBI-TAE, HAP, mHAP-2, six & twelve score. Detailed information about the model's characteristics and their performance is presented in Table 1.

**Table 1:** Details of models analyzed in the study.

Model name	Characteristics	No. patients	No. variables	C index	IBS	No. patients	No. variables	C index	IBS
(a) Radiomics (RSF)		OS				PFS			
P1	Arterial phase radiomics features with p < 0.05 from the univariate analysis	236	49	0.616	0.154	114	54	0.674	0.153
P2	Portal phase (analogous as above)	239	77	0.635	0.157	113	48	0.663	0.150
P3	Delayed phase (analogous as above)	198	67	0.640	0.162	90	51	0.690	0.152
Rad-Select	Selected features from all CT phases	186	193	0.629	0.158	89	153	0.713	0.158
Rad-Diff	Variable differences (between phases 1–2 or 2–3)	194	48	0.627	0.155	89	56	0.704	0.148
(b) Clinical (Cox PH)		OS				PFS			
TAB-model	All clinical variables from pre-TACE assessment	239	16	0.633	0.161	113	16	0.500	0.200
HAP [19]	Albumin < 36 g/dl; AFP > 400 ng/ml; Bilirubin > 17 umol/l; Tumor size > 7 cm	252	4	0.645	0.152	116	4	0.495	0.171
mHAP-2 [20]	HAP criteria + Tumor number ≥ 2	252	5	0.644	0.144	116	5	0.575	0.153
ALBI-TAE [22]	ALBI grade. AFP > 200 ng/ml; Up-to-11 criteria	252	3	0.651	0.151	116	3	0.562	0.159
Six & twelve score [21]	Largest tumor diameter; Tumor number	252	2	0.613	0.156	116	2	0.630	0.149

RSF, random survival forest; IBS, integrated Brier score; OS, overall survival; PFS, progression free survival; AFP, alfa-fetoprotein.

#### Radiomics models

For feature selection, we conducted univariate significance analyses using score tests. We determined the p-value thresholds by following the procedure detailed in separate publication^26^, and we enriched this approach with manual adjustments to account for various characteristics of individual modeling problems, such as discrepancies in the number of observations for OS and PFS, as well as the fraction of missing data. Features selected from the univariate analysis were included in the final radiomics models. A random survival forest (RSF) algorithm was used to deploy radiomics models. Overall, we have created ten models for OS and PFS prediction. Specifically, P1-3 models were generated using radiomics features from a single CT phase. Additionally, we created the Rad-Select model by combining the most significant features from different CT phases, and the Rad-Diff model that utilizes the differences between corresponding variable values extracted from subsequent CT phases, specifically between P1-P2 or P2-P3 (Table 1a).

#### Clinical models

We did not conduct feature selection on the clinical variables for this model, instead we generated a TAB-model incorporating all pre-TACE 16 clinical variables (listed in Supplementary Table 2). This model deployment was performed using the Cox proportional hazard (Cox PH) model and a cohort of patients for whom clinical information was complete. The TAB-model served as the main reference point for comparing the performance of radiomics models (summary presented in Table 1b).

### Statistical analysis

Statistical analyses were carried out using the R software (R Core Team 2023; R Foundation for Statistical Computing) and Python software (Python Software Foundation, version 3.11.1). Additionally, we used mlr3proba R package for modeling and performance evaluation and survex R package for model exploration and creating explanations^27,28^. In the process of building predictive models, RSF was employed for radiomics models, while Cox PH was used for clinical ones. For models’ performance evaluation, C-index and integrated Brier score (IBS) were used. The reported scores for OS models were obtained through 10 repetitions of eightfold cross-validation. In the case of PFS models, due to the limited dataset, we conducted evaluations using 10 repetitions of fivefold cross-validation. To compare distribution of performance metrics between the models, the Kruskal–Wallis and Mann–Whitney–Wilcoxon test were employed. The Pearson correlation coefficient was utilized to assess the linear correlations between the variables.

### Ethical approval and informed consent

The need for ethical approval and informed consent were waived for this study by the Bioethics Committee at the Medical University of Warsaw due to its retrospective study design.

## Results

The study enrolled a total of N = 252 patients while Table 2 displays the detailed baseline characteristics of the entire cohort. The percentage of patients with missing data for at least one of the clinical variables in the dataset set was 5.6%.

**Table 2:** Baseline patients’ characteristics

Variable	No. of patients	(%) or median, (range)
Age, years (median)	66.5 (28–86)	
Gender, female (%)	52 (21%)	
*Aetiology of chronic liver disease*		
Viral	113	45
Alcoholic	81	32
Mixed	20	8
Other	38	15
*Child turcotte pugh class*		
A	226	90
B	26	10
AFP		
< 200 ng/mL	183	73
≥ 200 ng/mL	69	27
Albumin (g/L)	4.0	1.1–5.2
Creatinine (mg/dl)	0.91	0.49–2.79
Total bilirubin (umol/L)	0.83	0.22–7
INR	1.15	0.88–1.85
ALT (IU/L)	43	9–800
*Number of lesions*		
1	162	64
2	54	21
3	20	8
> 3	15	6
Lesion size (cm, median)	45	10–180
< 3 cm	54	21
3–5 cm	87	35
> 5 cm	111	44
*HAP score*		
A	84	33
B	96	38
C	54	21
D	18	7
*ALBI TAE*		
A	95	38
B	114	45
C	37	15
D	6	2
Number of TACE procedures	2.0	1–10
2	123	49
3	34	13
> 3	93	38

AFP—alfa-fetoprotein; INR—international normalized ratio; ALT—alanine transaminase.

### Overall survival prediction

**Figure 2:**
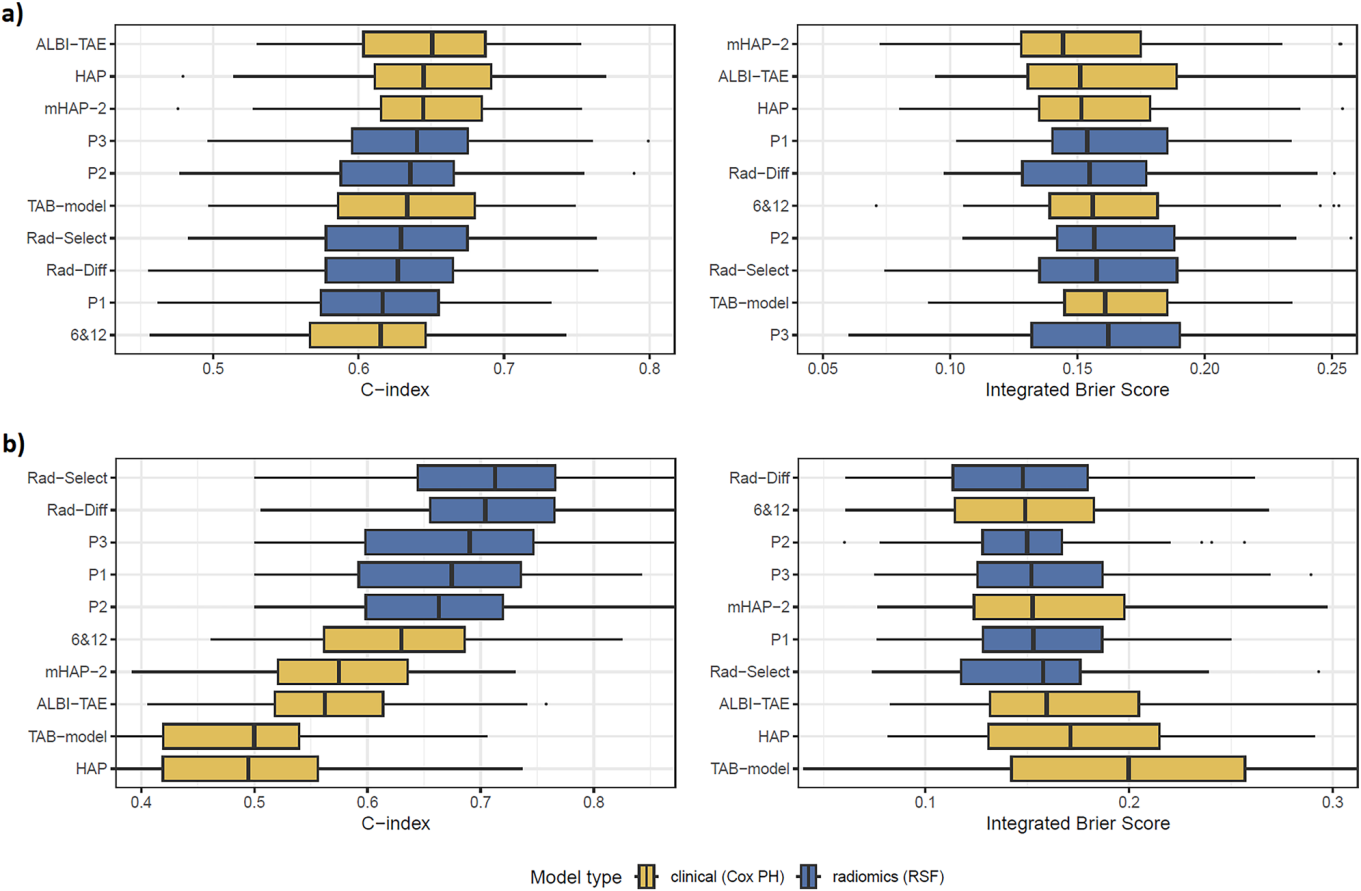
Metrics of models’ performance; (**a**) for overall survival and (**b**) for progression free survival.

At the time of the analysis, a total of 177 out of 252 patients (70%) in the study cohort had died. The median OS for the entire cohort was 109 weeks (95% CI 93.1–143), with a range from 1.14 to 377.71 weeks. Figure 2a presents the performance for OS prediction of the five radiomics and five clinical models.

There was no significant difference between the models P1, P2, P3, Rad-Select, Rad-Diff and TAB-model (p = 0.25 and p = 0.67 for C-indexes and IBS, respectively). The performance of the P1 model did not differ significantly from the 6&12 score (p = 0.70 and p = 0.79 for C-indexes and IBS, respectively), while the P3 had significantly higher C-index (p = 0.0091). The best overall performance was observed for ALBI-TAE, HAP and mHAP-2, however they showed only a slight improvement over the P2 and P3 radiomics models (C-indexes of 0.651–0.644 compared to 0.640–0.635 [p = 0.27], and IBS of 0.152–0.144 compared to 0.162–0.157 [p = 0.22], respectively). The best performance among the radiomics models was achieved by the P3 model with the highest C-index of 0.640 and the P1 model with the lowest IBS of 0.154.

When analyzing the results for a single model and different subsets of the data, the best performance was obtained for models trained on non-radiomics clinical data with selected radiomic features. However, the additional benefit was not statistically significant. Moreover, adding radiomic variables did not significantly improve the performance of the evaluated clinical models.

### Progression free prediction

Among the entire cohort, 116 (47%) achieved a LR-nonviable TACE response. Among those, 61 out of 119 (51%) experienced disease progression over a median follow-up period of 110 weeks (95% CI 99–124). Box plots showing the performance of the models for PFS prediction are presented in Fig. 2b.

Overall, the radiomics models outperformed the clinical ones in terms of median C-indexes (0.713–0.663 compared to 0.630–0.495, respectively). There was significant difference between values of this metric for models P1, P2, P3, Rad-Select, Rad-Diff and TAB-model (p = 0.00093). The highest C-index of 0.713 was observed for the Rad-Select model and it was significantly better than best clinical model of 6 & 12 (p = 0.00041). In contrast, median integrated Brier scores for radiomics and clinical models are similar for both types of models (0.158–0.148 compared to 0.200–0.153, respectively). However, there was significant difference between the radiomics models and TAB-model (p = 0.022). The lowest IBS was observed in Rad-Diff and 6 & 12 models (0.148 and 0.149, respectively, p = 0.74).

### Explainable analysis

**Figure 3:**
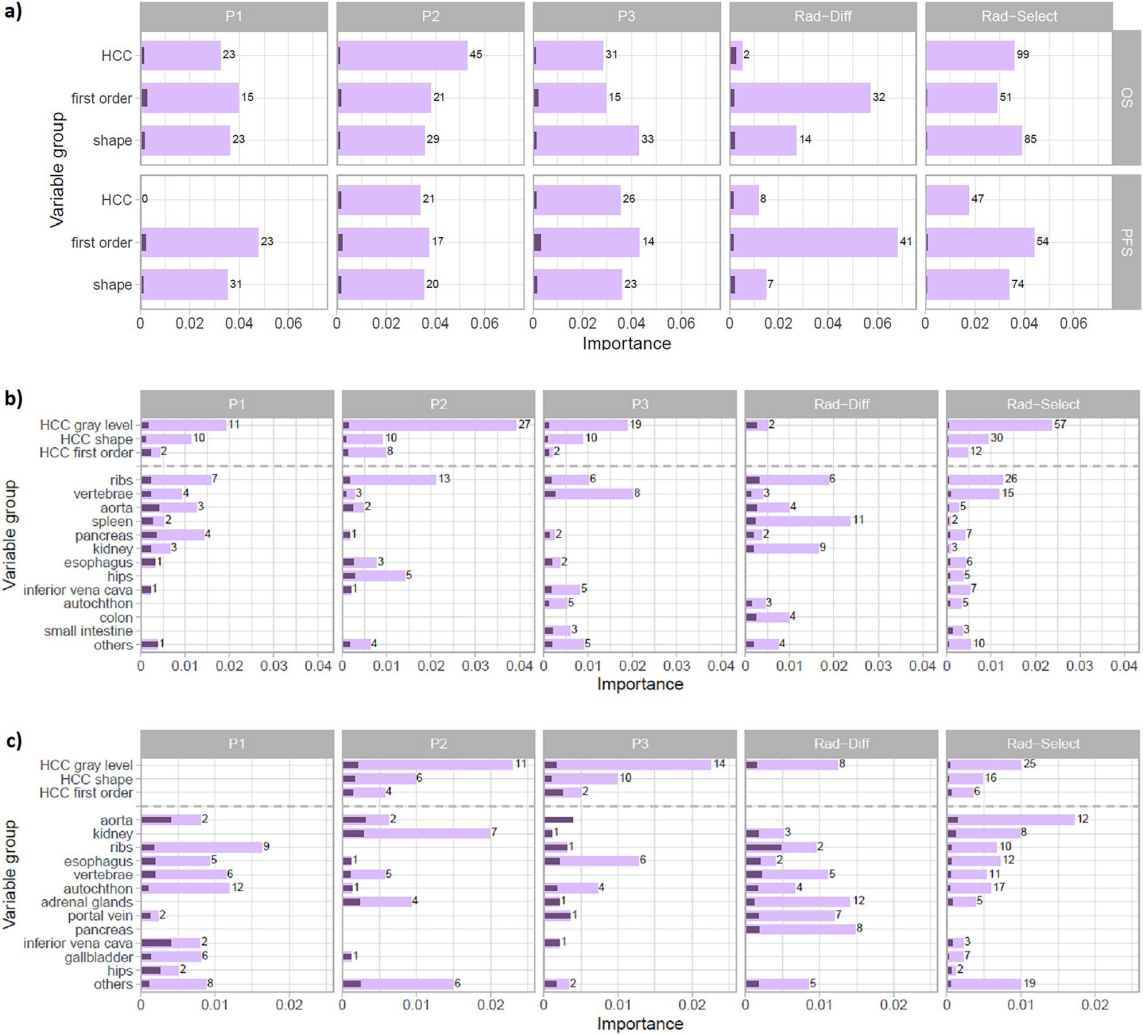
Analysis of features’ importance for radiomics models; (**a**) aggregated variables’ significance, the first-order and shape-based bars on a plot represent the importance of features for non-HCC volumes, while the HCC bar encompasses the aggregation of all feature groups; (**b**) aggregated feature importance across different volumes of Interest (VOIs) for overall survival (OS); (**c**) aggregated feature importance across different VOIs for progression free survival (PFS); light bars represent the total importance of the group; dark bars indicate the average importance per variable; the numbers indicate the number of variables from a given group in this model.

The aggregated importance of the variables included in radiomics models is presented in Fig. 3a. The significance of first order or shape-based features of non-HCC VOIs was higher than the aggregated importance of features of the largest HCC lesion in every radiomics model with the exception of the P2 model.

However, when examining VOIs separately, it was generally observed that features derived from the largest liver tumor, particularly gray level features, held the highest significance in predicting both OS and PFS (Fig. 3b and c). The features of the largest liver tumor, especially those related to shape and gray level, were consistently included in the P1-3 models for both OS and PFS. Notably, P1-PFS was an exception, as none of the features related to HCC proved to be significant for this model. Overall, the largest liver tumor features were the only variables among the 20 most frequently repeated features in models P1-3. It's worth mentioning that multiple gray-level HCC features proved to be important for P2-OS (that utilize portal CT phase features), aligning with the highest correlation observed between automatic and manual segmentation in portal CT phase examination (as presented in Section "Largest liver tumor diameter".).

Importantly, the relevance of non-tumoral VOI features was significant in every radiomics model, while their cumulative importance was higher than features extracted from the largest liver tumor (Fig. 3a). Bone features, which included VOIs for ribs and vertebrae, demonstrated significance across a substantial portion of the models (Fig. 3b and c). Notably, for OS prediction, the significance of hips features was significantly pronounced in the P2 model, given their inclusion mainly in this phase of the study. Of note, there was relatively high significance of features of aorta VOI in the P1-OS (that uses the arterial phase CT with optimal contrast of the vessel lumen). Conversely, there was either a lack of significance or minimal significance of aorta features in the subsequent CT phases.

Turning to the Rad-Diff models for OS and PFS, differences between individual study phases held particular significance for non-HCC first-order features while showing lesser importance for the other groups (Fig. 3a). Especially differences among radiomics features for the spleen and kidneys held considerable importance for the Rad-Diff-OS model, with their isolated values being of lesser importance for P1-3-OS.

### Examples of in-depth variable analysis

#### Largest liver tumor diameter

The correlation between the largest axis length, as determined through automated tumor segmentation, and the maximal axial diameter reported by the radiologist was strong for the portal and delayed phases (r = 0.76 and 0.73, p < 0.01 for both, respectively), and moderate for the arterial phase (r = 0.68, p < 0.01). Importantly, the maximal largest tumor diameter reported by the radiologist was one of the top two variables important for OS prediction.

#### Inferior vena cava

**Figure 4:**
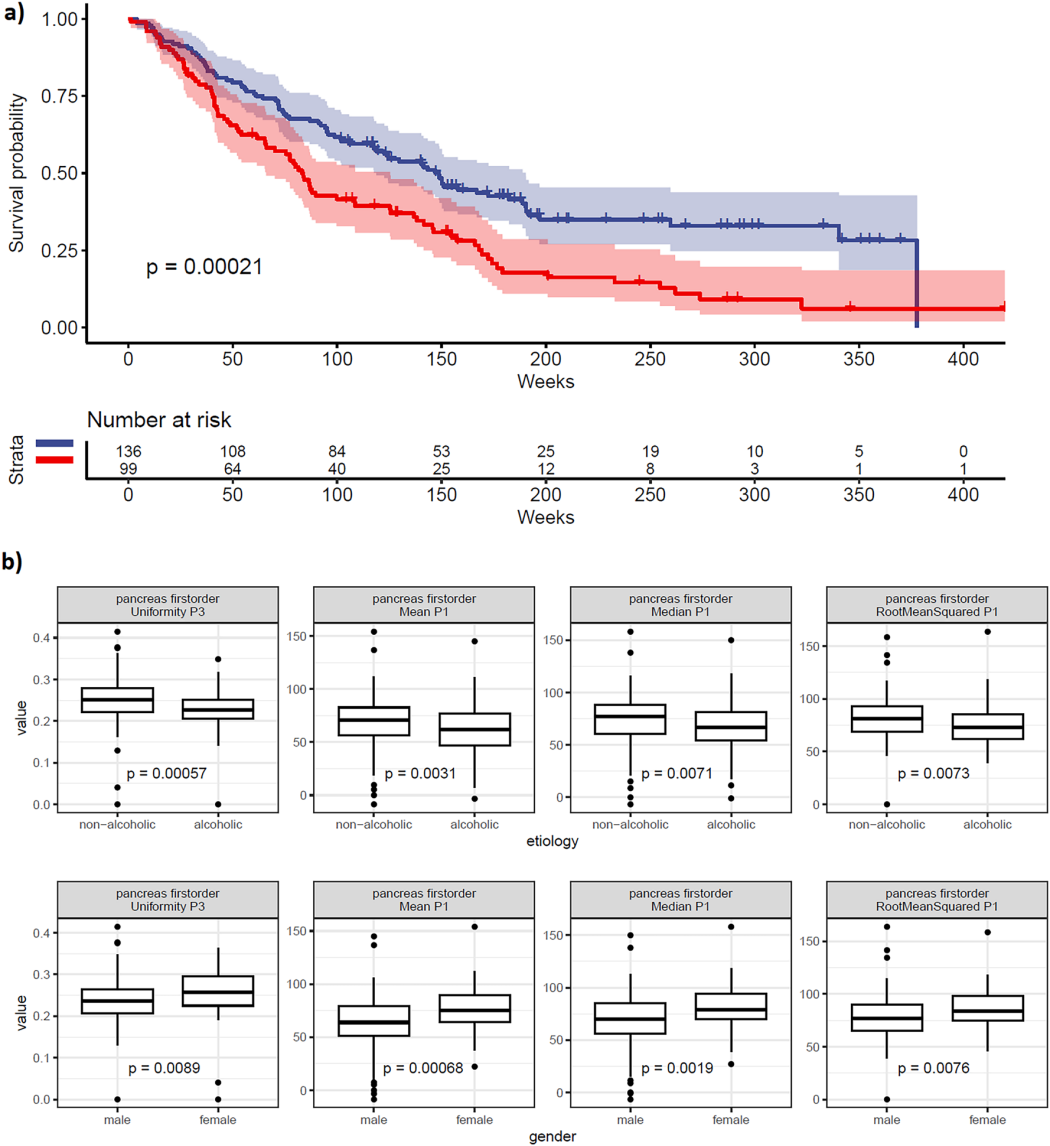
Examples of in-depth variable analysis; (**a**) Kaplan–Meier plot showing the OS based on the _Max2DDiameter of the IVC. The optimal cut-off point was determined using the Contal & O’Quigley procedure^29^; (**b**) selected radiomics features of the pancreas compared between alcoholic cirrhosis and nonalcoholic cirrhosis, as well as between males and females—the nominal level of significance for correlation with the Bonferroni correction was set to *p* < 0.0071.

Shape-based features of the inferior vena cava (IVC) were significant predictors in the P1-3 and Rad-Select models. Specifically, _Max2DDiameter was significant for P1 and P2, while _MeshVolume, _SurfaceArea, and _VoxelVolume were significant for P3. These findings prompted us to generate Kaplan–Meier curves for the _Max2DDiameter of the IVC during the P1 phase, which revealed a significant impact of this covariate on OS prognosis (Fig. 4a).

#### Pancreas

Selected first-order and shape-based features of the pancreas were significant predictors in the P1-3 models, all of which were included in the Rad-Select model. We observed significantly higher values for these features in males and individuals with alcoholic cirrhosis, which were significant predictors of shorter OS in univariate analysis. Figure 4b presents the values of selected radiomics features for the pancreas in males versus females and alcoholic cirrhosis versus nonalcoholic cirrhosis. Beside the observed significant differences in the values of pancreatic features, the general trend without reaching statistical significance was also visible for other features of this VOI.

## Discussion

In this study, we propose a novel ML radiomics approach for multifactorial task of prediction of TACE outcomes in HCC patients. As far as we know, this is the first study to predict TACE outcomes using a fully automated deep-learning workflow for multiple organ VOIs segmentation. In contrast to other studies, we propose an approach using radiomics features extracted from various organ VOIs, beyond the HCC lesions. Completing this task using a manual segmentation would be too time-consuming to be feasible. Our pipeline utilized a three-step process. First, we used a pre-trained deep learning model for VOI segmentation. This allowed for the extraction of multiple radiomics features from various organ VOIs in each of the CT phases. Second, ML models for OS and PFS prediction using solely image-derived radiomics features were deployed. The aim was to fully leverage the information encoded in distinct organ volumes, mimicking standard multifactorial clinical evaluations. Third, as interpretation is important to make it acceptable to clinicians, we used XAI to generate explanations for the proposed models.

We have found that radiomics RSF models demonstrate comparable performance to analogous Cox PH multivariate models that use clinical data for OS prediction. However, the performance of the radiomics approach was slightly lower when compared to state-of-the-art clinical models like HAP and ALBI-TAE. The absolute performance differences were, in fact, very low and the clinical significance is probably negligible. In terms of PFS prediction, the proposed radiomics approach outperformed every clinical model tested in the present study. However, this finding must be treated with caution, as the PFS analysis was performed on a limited subset of patients that achieved a favorable (LR-nonviable) TACE response. Nevertheless, the exceptional performance for PFS prediction encourages further research and warrants validation in external cohorts.

Another important findings emerge from the XAI analysis. Overall, imaging features extracted from VOIs of the largest liver tumors have consistently proven to be the most relevant variables in our models, in line with previous radiomics studies^5,8,30–32^. However, the most intriguing aspect of our study is the relatively high importance of features derived from non-tumoral VOIs. These results suggest that factors beyond the liver tumor can significantly impact long-term TACE outcomes. In other words, our findings indicate that radiomic analysis, which encompasses non-tumoral regions, has the potential to enhance a comprehensive and accurate prediction of patient prognosis. Notably, there is substantial clinical potential in translating our proposed multi-VOI approach into other clinical scenarios, such as general oncology or lung disorders.

An explanatory analysis of the models revealed several additional interesting findings. The interpretation of a strong correlation between the largest HCC diameter assessed by radiologist and the segmentation algorithm is straightforward and allows for relatively easy model explainability. However, we observed a lack of evident correlations between radiomics features and laboratory examinations. This suggests that a significant fraction of the performance of radiomics models may be due to their ability to predict without the need to rely on proxies for clinical variables. For example, IVC diameter is one of the interesting variables that was suggested as a significant predictor for OS. Our results indicate that a larger IVC diameter was an unfavorable prognostic factor for OS, which aligns with domain knowledge. Notably, the width of the inferior vena cava is not included in routine clinical evaluation, but it’s diameter may be correlated with several factors, including the breathing phase (inhalation or exhalation), heart function, liver disease, and the patient's level of hydration^33^. Regarding the breathing phase, CT scans are typically performed during a breath-hold (when the IVC is usually slightly collapsed or smaller). Patients in poorer clinical condition may have difficulty performing this maneuver effectively, potentially resulting in a higher IVC maximal diameter. Similarly, in cases of right ventricular failure, the IVC tends to be wider^34^. Another factor to consider is liver disease, where in theory, the IVC may be wider in more advanced cirrhosis^35^. Similarly, the correlation between radiomics feature values and male gender as well as alcoholic liver disease may partially explain the importance of these features in the context of OS prediction^36,37^. However, it is worth noting that the individual importance of these features was relatively low, that suggest that the radiomics models demonstrated their predictive strength by including numerous seemingly unrelated features from multiple VOIs. Notably, to aggregate our explanatory findings, we utilized the permutation feature importance method, presenting aggregated feature importance for each radiomics model. A detailed, in-depth XAI analysis, e.g. employing precise Shapley values to present the most important features for specific VOIs and models, could serve as an intriguing foundation for future research. Although falling beyond the scope of the present study, this approach may facilitate the discovery of potential novel radiomics predictors and testing their significance in future studies.

The rationale for a multi-VOI approach can be found in previous studies. Traditionally, outcome prediction models in HCC patients primarily rely on tumor burden (e.g., number and size of foci) and the severity of underlying liver disease. In turn, most AI models focus on radiomics analysis of tumoral VOIs. However, previous studies have suggested that other volumes can be of clinical significance. For instance, in a recent study, volumetric liver and spleen CT data was employed to predict the severity of hepatic fibrosis in patients with chronic liver disease^13^. Another example of applying AI methods in liver diseases is the recent model for CPS prediction where the authors used liver parenchyma and hepatic vessels VOIs to predict CPS with reasonable performance^14^. This highlights the significance of features derived from VOIs beyond liver tumor that may contain complementary information valuable for outcome prediction.

While it is challenging to directly compare the obtained results with previous radiomics analyses, it is worth mentioning other studies that demonstrate the potential of radiomics in predicting outcomes in TACE patients^38^. For example, in a study by Xiang-Pan Meng et al. radiomics features were extracted from manually segmented tumoral and peritumoral regions in CT images from N = 162 patients^39^. The radiomics model included six features and achieved a C-index ranging from 0.664 to 0.669, whereas the combined radiomics-clinical model demonstrated a higher C-index ranging from 0.7 to 0.73. The proposed radiomics-clinic model outperformed seven existing prognostic models, including the HAP score and its variations. The authors suggested that combining radiomics features with clinical data could improve performance for OS prediction in TACE patients. Importantly, the authors suggested that radiomics analyses using multiphasic enhancement CT images can be superior to single-phase imaging as they can provide more comprehensive information. However, this approach is limited by the laborious task of tumor segmentation across each phase. Here, we overcame this limitation by implementing a fully automated segmentation pipeline.

The other study aimed to develop radiomics nomograms for OS and PFS in N = 60 patients with more advanced BCLC-C patients with HCC treated with TACE and apatinib^40^. Imaging features were extracted from manually segmented tumor VOIs during both the late arterial and portal venous phases. Again, the combined clinical-radiomics nomograms outperformed the radiomics model in predicting both OS and TTP. However, the authors did not compare a radiomics-based approach to clinical models in the study. In contrast to our study, the performance of the radiomics approach was lower for predicting PFS compared to OS. This may be potentially explained by the different performance of the models in more advanced HCC stages. It would be interesting to validate our approach using more advanced HCC cohorts.

Another radiomics study aimed to enhance the prediction of treatment response to TACE in N = 61 HCC patients^41^. In contrast to our study, the authors used post-TACE CT examinations while the tumor segmentations were performed semi-automatically with manual correction. Importantly, the radiomics-clinical model performed well for OS prediction with a C-index of 0.67, while the single feature radiomic model had a C-index of 0.6.

Recently, a comprehensive model was developed, integrating contrast-enhanced CT features and clinical factors to predict OS in a subset of N = 261 HCC patients undergoing TACE ^42^. After adjusting for clinical variables, the model combining deep learning, radiomics and clinical features proved to be the most effective predictor of OS in the multivariate analysis. This finding suggests the potential value of the comprehensive model in predicting OS. In our study, we solely utilized an ML model, but it would be interesting to combine it with a deep learning-based approach to evaluate if it can offer superior performance for OS prediction.

In all four studies, the combined radiomic-clinical models outperformed clinical models for OS prediction. In contrast, in our study, the addition of radiomics features to clinical data did not result in an improvement in the performance of the prediction models (details in Supplementary Figs. 1 and 2). To ensure our results are comparable to existing studies, we selected the CoxPH for clinical models for its well-established role in clinical research. Additionally, we chose the RSF model for its ability to handle high-dimensional time-dependent data and its proven effectiveness in radiomics studies. Our approach, employing both CoxPH and RSF, aimed to bridge traditional clinical methodologies with advanced radiomics techniques, facilitating comparative analysis of those two different outcome prediction approaches.

The solutions proposed in our study address several significant issues. First, we introduce an approach that doesn't require domain knowledge in radiology. This is highly significant because it not only aids in medical decision-making but also broadens the potential utility of CT radiomics in scenarios where clinical data may be limited or unavailable.

Second, the automated segmentation utilized in our study greatly reduces segmentation time. Importantly, the overall Dice score for the automated multiple organ segmentation achieved by the model was 0.943, with a 95% CI ranging from 0.938 to 0.947, as reported by Wasserthal et al.^18^. This approach enhances model scalability since it can be readily applied to large datasets. Regarding the radiomics features of tumors, we employed a simplification approach, focusing on the analysis of the largest HCC lesion. This method aligns with prior study, that have validated the feasibility of assessing the largest lesion in survival analysis after TACE^43^. The automated approach allows for the segmentation of each phase of the examination separately. However, this unsupervised pipeline may introduce potential biases. In our experience, the automated segmentation of HCC lesions, particularly those which are well-defined and encapsulated, has shown very good visual performance. Conversely, we acknowledge that certain HCC types, characterized by infiltrative growth patterns or the presence of satellite foci, present significant challenges for segmentation. These issues arise from the ambiguous appearance on imaging and the blurry boundaries between lesions and liver parenchyma, potentially leading to biased radiomics feature extraction from the liver tumor. Future studies should evaluate whether manual segmentation of HCC could enhance the performance of our approach, despite the trade-off of time consuming process. Of note, our initial observations indicate a very high visual concordance between the outcomes of deep learning segmentation and the assessments of expert radiologists, VOIs of internal organs. This substantial alignment was also expressed in the metrics supplied by the authors of the "TotalSegmentator" model we employed^18^.

Despite using a set of predefined VOIs, our approach is adaptable and allows for the easy inclusion of additional VOIs that could potentially improve model performance. For example, Mohammadi et al. show that the tumor microenvironment has diagnostic potential in discriminating benign and malignant lesions using machine learning models for medical image analysis^44^. The findings emphasize that radiomics characteristics from expanded contours of the tumor periphery might improve the diagnostic performance of AI models. Integrating the tumor microenvironment into our model could improve its performance and offers a promising avenue for further analysis.”

Moreover, multiphase CT imaging enables the analysis of radiomics feature variability over time, specifically across successive phases of the study. This represents a novel approach that has not been previously explored. Notably, we observed significant changes in the values of radiomics features over subsequent CT phases and their relevance in predicting TACE outcomes, particularly in the case of the spleen and kidneys. In contrast, the significance of features derived from individual phases was less pronounced in this context. This novel direction in radiomics development, facilitated by the utilization and advancement of automatic segmentation tools, offers new opportunities for research in the field of radiomics. Notably, the non-contrast phase yielded suboptimal results within our methodology. In our study, the low overall performance of the models trained on the non-contrast phase led us to exclude the native phase from the presented results. This was likely attributable to suboptimal organ segmentation in non-contrast CT. This contrasts with a recent study in which a combination of non-contrast CT radiomics and clinical variables proved to be effective in predicting overall survival (OS) in HCC patients following TACE^45^.

### Limitations

First, we have not conducted a robustness analysis of the radiomics features used in the models. It is possible that some of these features may not exhibit consistency and repeatability across different datasets. Future studies involving external validation cohorts may help clarify these issues.

Second, radiomics models were trained using a relatively small dataset and there is room for further performance improvement, particularly with the prospect of incorporating more imaging data in the future. Given the large number of predictors used in our models and the high dimensionality of our feature set, there is a risk of overfitting, and thus these results should be considered preliminary. This particular aspect may have an impact on the prediction of PFS, therefore those results should be approached with caution and should undergo validation in a larger cohort. To address this, we used k-fold cross-validation approach that maximizes the utilization of available data without sacrificing information. Nevertheless, it can pose challenges when comparing model parameters across different studies. To overcome this issue, we have compared different models using our dataset and the performance of HAP or ALBI scores in our study aligned with those reported in literature^21,46^.

Third, the choice of variables was based on simplified approach using significant predictors from the univariate analysis. Our experiments with LASSO and Boruta framework resulted in models exhibiting significantly lower performance. This could potentially be attributed to the high dimensionality of predictors and the diversity of their groupings (VOIs, study phases or types of radiomic features). Interestingly, it appears that in the presence of a high noise-to-signal ratio, conventional feature selection techniques outperform methods that are based on machine learning models. Future studies would benefit from evaluating alternative approaches, such as Coe-Thr-Lasso method, that facilitate a more holistic analysis of variable importance^47^.

Fourth, the utilization of different CT scanners may potentially impact the extraction of imaging features and consequently influence the performance of the model. However, it is important to note that this scenario represents real-life clinical practice, where patients may undergo check-up examinations in outpatient settings outside of tertiary health centers. On the other hand it can be beneficial in minimizing the risk of the incorporation of non-relevant features in the model. It must be clearly stated that no CT image normalization or filtering was applied prior to segmentation and feature extraction in our study. This may be particularly important for the reliability of gray-level feature estimates and, to a lesser extent, shape-based and first-order features^48,49^. However, given the preliminary nature of our study and the lack of standardization in preprocessing steps, we opted for a straightforward exploratory analysis. The predictive performance of our approach could potentially be improved by utilizing image preprocessing steps such as normalization or filtering.

Finally, the overall implementation of radiomics models is relatively complex and not easy to deploy on resource-limited hardware. However, we utilized an open-source segmentation model, whose user-friendly version is available online^50^. By combining this approach with recently released easy-to-use radiomics tools, such as “autoradiomics”, it becomes easy for other research teams to deploy and test this approach on external datasets^51^.

In conclusion, we demonstrated the potential of a novel multi-VOI radiomics approach for TACE outcome prediction. The proposed pipeline eliminates the need for radiologist input and addresses the issues associated with time-consuming manual VOI segmentation. Furthermore, this study underscores the clinical significance of VOIs beyond tumor regions, which can be utilized to extract clinically relevant information. This suggest that radiomics analysis should not be limited to the tumor itself but should consider other organ VOIs as well. Such a holistic approach may lead to more accurate predictive models and improved patient care of HCC patients. Moreover, these findings may have broader applicability in various clinical scenarios, butwarrant further validation in prospective or external cohorts.

### Supplementary Information

Supplementary Information.

## Data Availability

The CT dataset are not currently publicly available due to privacy regulations. Remaining data analyzed during the current study can be obtained from the corresponding author on reasonable request.
