# Supplementary material for: A novel radiomics approach for predicting TACE outcomes in hepatocellular carcinoma patients using deep learning for multi-organ segmentation
Source: Sci Rep. 2024 Jun 26;14:14779. doi: 10.1038/s41598-024-65630-z (PMC11208561; doi:10.1038/s41598-024-65630-z)
Supplement: Supplementary file 1 — Supplementary Information. [file 41598_2024_65630_MOESM1_ESM.docx]

**A Novel Radiomics Approach for Predicting TACE Outcomes in Hepatocellular Carcinoma Patients Using Deep Learning for Multi-organ Segmentation**

**Supplementary Materials**

Authors: Krzysztof Bartnik*^1^, Mateusz Krzyziński^2^, Tomasz Bartczak^2^, Krzysztof Korzeniowski^1^, Krzysztof Lamparski^1^, Tadeusz Wróblewski^3^, Michał Grąt^3^, Wacław Hołówko^3^, Katarzyna Mech^4^, Joanna Lisowska^4^, Magdalena Januszewicz^1^, Przemysław Biecek^2^

1. Second Department of Radiology, Medical University of Warsaw, Warsaw, Poland, Banacha 1a st.
2. Faculty of Mathematics and Information Science, Warsaw University of Technology, Warsaw, Poland, Koszykowa 75 st.
3. Department of General, Transplant and Liver Surgery, Medical University of Warsaw, Warsaw, Poland, Banacha 1a st.
4. Department of General, Gastroenterological and Oncological Surgery, Medical University of Warsaw, Warsaw, Poland, Banacha 1a st.

*Corresponding author: Krzysztof Bartnik MD, PhD, [krzysztof.bartnik@wum.edu.pl](mailto:krzysztof.bartnik@wum.edu.pl), cell phone: +48 606 745 140; Banacha 1a st, 02-097 Warsaw, Poland.

**Short title:** Multi-volume radiomics for TACE outcome prediction.

**Funding:** This research was carried out with the support of the Laboratory of Bioinformatics and Computational Genomics and the High Performance Computing Center of the Faculty of Mathematics and Information Science Warsaw University of Technology.

**Competing interests:** The authors declare no competing interests.

Contents

[**Supplementary Table S1.** Patients’ inclusion and exclusion criteria. 2](#_Toc161674731)

[**Supplementary Table S2.** Summary of evaluated clinical variables and extracted radiomics features. 3](#_Toc161674732)

[**Supplementary Table S3.** Details of computed tomography phase acquisition and scanners. 4](#_Toc161674733)

[**Supplementary Table S4.** Table of the performance comparisons with other similar studies discussed in the manuscript. 5](#_Toc161674734)

[**Supplementary Table S5.** List of volumes of interest included in the final analysis. 6](#_Toc161674735)

[**Supplementary Materials S6.** Comparative analysis of radiomics-based models versus combined radiomics and clinical data models. 7](#_Toc161674736)

# **Supplementary Table S1 and patient selection flow diagram.** Patients’ inclusion and exclusion criteria.

| **Inclusion Criteria** | **Exclusion Criteria** |
| --- | --- |
| 1) Patients with at least one confirmed HCC lesion | 1) Patients who underwent liver transplantation, resection, or ablation before inclusion or during follow-up period |
| 2) Patients undergoing TACE | 2) Patients with a history of malignant neoplasm other than HCC |
| 3) Patients with good liver function (CPS A or B) | 3) Patients with uncontrolled functional or metabolic disease prior to the procedure or during follow-up period |
| 4) Patients with available multiphase imaging within 90 days before TACE session |  |


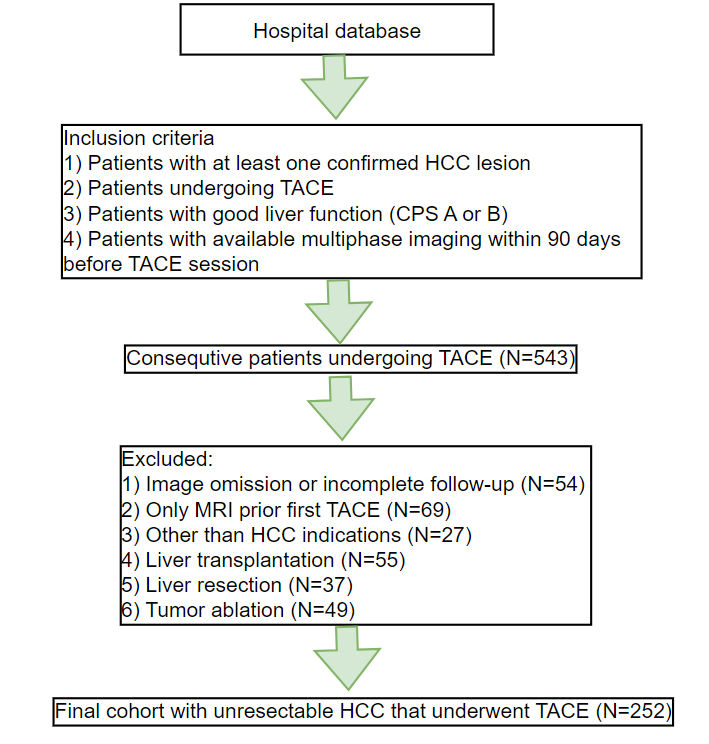


# **Supplementary Table S2.** Summary of evaluated clinical variables and extracted radiomics features.

| **Clinical variables (N=16)** | **Description** |
| --- | --- |
| Age | Age of the study participants |
| Sex | Gender of the study participants |
| Underlying Etiology of Chronic Liver Disease | HBV, HCV, Alcoholic, Mixed, NASH or Cryptogenic |
| Number of HCC Lesions | Count of HCC lesions |
| Lesion Location | Left or right liver lobe |
| Lesion Diameter | Maximal diameter in axial plane (portal phase CT) |
| LI-RADS Category | LI-RADS classification of lesions |
| Initial Treatment Response Category | Based on LI-RADS treatment response algorithm |
| BCLC stage | Barcelona-Clinic Liver Cancer Stage |
| Child-Pugh Score (CPS) | Child-Pugh Score |
| Serum Levels: |  |
| Total Bilirubin | Serum level of total bilirubin |
| Albumin | Serum level of albumin |
| Creatinine | Serum level of creatinine |
| INR | International normalized ratio |
| AFP | Serum level of α-fetoprotein |
| ALT | Serum level of alanine aminotransferase |
| **Radiomics features** | **Number of Features in each category** |
| **Shape-Based (for all VOIs)** | 14 |
| Descriptors of 3D size and shape |  |
| **First-Order (for all VOIs)** | 18 |
| Distribution of voxel intensities |  |
| **Gray-Level Radiomics Features (only for largest liver tumor)** | 75 |
| Gray-level co-occurrence matrix | (24) |
| Gray-level dependence matrix | (14) |
| Gray-level size zone matrix | (16) |
| Gray-level run length matrix | (16) |
| Neighbouring gray tone difference matrix | (5) |

# **Supplementary Table S3.** Details of computed tomography phase acquisition and scanners.

| **Study Phase** | **Bolus Tracking Technique** | **Number of Exams** | | | | |
| --- | --- | --- | --- | --- | --- | --- |
| Late Arterial | 15-30 seconds post-bolus | 236 | | | | |
| Portal Venous | 60-75 seconds post-injection | 239 | | | | |
|  |  |  | | | | |
| Delayed | 4-5 minutes | 198 | | | | |
| **CT system** | **Manufacturer and Location** | **Number of Exams** | | | | |
| Optima CT600 | General Electrics HealthCare, Boston, 5 Necco St, United States | 170 | | | | |
| Somatom Xceed | Siemens Healthcare; Aktiengesellschaft, Werner-von-Siemens-Straße 1 80333 Munich Germany | 36 | | | | |
| Ingenuity Core | Philips Healthcare; Eindhoven, High Tech Campus 5, Netherlands | 23 | | | | |
| Aquilion One | Toshiba Medical Systems Corporation, 1385 Shimoishigami, Otawara City, Tochigi Prefecture | 23 | | | | |
| **CT parameter** | **Values** |  | | | | |
| Slice thickness per phase (median) | 1.5 mm for arterial; 1.5 mm for portal; 1.25 mm for delayed | | |  | | |
| Axial dimension | Matrix of 512x512 (for 99,4% of CT series; N=4 series were 768x768) | | | |  | |
| Tube voltage | 120 kVp (for 233/252 CT examinations), 100 - 140 kVp for remaining | | | |  | |
| Tube current | Automated modulation ranging from 250 to 300 mA | |  | | | |
| Reconstruction algorithm | Standard soft tissue kernel | | |  | | |
| Contrast enhancement | All images were obtained using intravenous contrast | | | | |  |
|  |  |  | | | | |
|  |  |  | | | | |
|  |  |  | | | | |

# **Supplementary Table S4.** List of volumes of interest included in the final analysis.

| **Index** | **Name of volume of interest** |
| --- | --- |
| 1 | liver_tumor |
| 2 | spleen |
| 3 | kidney_right |
| 4 | kidney_left |
| 5 | gallbladder |
| 6 | liver |
| 7 | stomach |
| 8 | aorta |
| 9 | inferior_vena_cava |
| 10 | portal_vein_and_splenic_vein |
| 11 | pancreas |
| 12 | adrenal_gland_right |
| 13 | adrenal_gland_left |
| 14 | vertebrae_L5 |
| 15 | vertebrae_L4 |
| 16 | vertebrae_L3 |
| 17 | vertebrae_L2 |
| 18 | vertebrae_L1 |
| 19 | vertebrae_T12 |
| 20 | vertebrae_T11 |
| 21 | vertebrae_T10 |
| 22 | esophagus |
| 23 | iliac_artery_left |
| 24 | iliac_artery_right |
| 25 | iliac_vena_left |
| 26 | iliac_vena_right |
| 27 | small_bowel |
| 28 | duodenum |
| 29 | colon |
| 30 | rib_left_10 |
| 31 | rib_left_11 |
| 32 | rib_left_12 |
| 33 | rib_right_10 |
| 34 | rib_right_11 |
| 35 | rib_right_12 |
| 36 | hip_left |
| 37 | hip_right |
| 38 | sacrum |
| 39 | autochthon_left |
| 40 | autochthon_right |
| 41 | iliopsoas_left |
| 42 | iliopsoas_right |
| 43 | urinary_bladder |

# **Supplementary Table S5.** Table of the performance comparisons with other similar studies discussed in the manuscript.

| **Authors** | **Study group** | **Cohort (training/testing)** | **Imaging modality and VOI** | **Model type** | **Performance in testing cohort** |
| --- | --- | --- | --- | --- | --- |
| Bartnik et al. (present study) | BCLC A-B | N=252 (OS)  N=116 (PFS)  (k-fold cross-validation) | Pre-TACE CT (multiphase), automated N=43 VOI (tumoral and internal organs) | Radiomics only | OS: C-index of 0.62-0.64;  PFS: C-index of 0.66-0.71 |
| Xiang-Pan Meng et al. | BCLC A-B | N=162 (108/54) | Pre-TACE CT (arterial and venous phase), manual segmentation of HCC and peritumoral VOI | Radiomics and radiomics-clinical | OS: C-index of 0.67 (0.56-0.79) for radiomics; C-index of 0.7 (0.62-0.82) for radiomics-clinical |
| Lingli Li et al. | BCLC C (apatinib +TACE) | N=60 (48/12) | Pre-TACE CT (arterial and venous phase), manual segmentation of HCC | Radiomics-clinical | OS: C-index of 0.745 (0.695-0.795);  PFS: C-index of 0.586 (0.543-0.628) |
| Simon Bernatz et al. | BCLC A-C | N=61 (42/19) | Post-TACE non-contrast CT, manual HCC segmentation | Radiomics and radiomics-clinical | OS: C-index of 0.6 for radiomics-only;  C-index of 0.67 for radiomics-clinical; |
| Zhongqi Sun et al. | BCLC-B | N=251 | Pre-TACE arterial phase CT, manual HCC segmentatnion | Radiomics-clinical | Combined deep learning, radiomics and clinical as independent risk factor in CoxPH analysis, HR 1.2 (1.03-1.4) |

# **Supplementary Figures.** Comparative analysis of radiomics-based models versus combined radiomics and clinical data models.


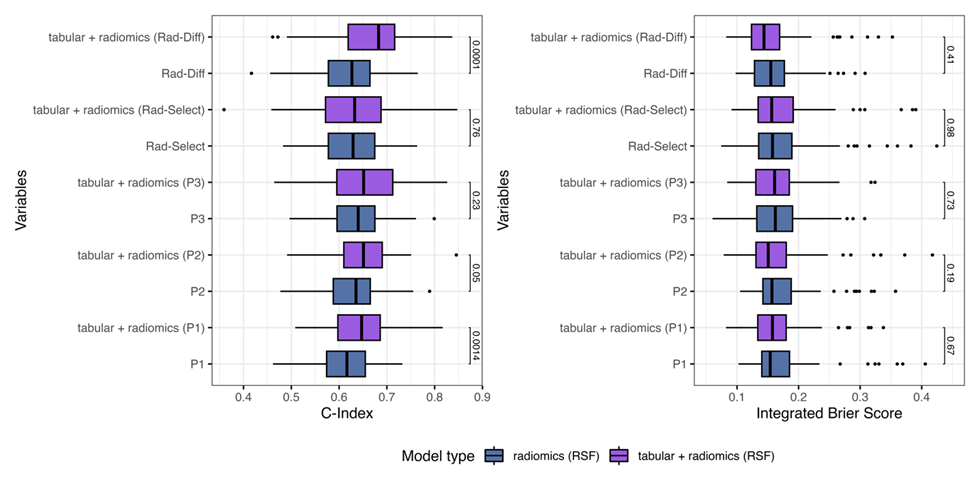


**Supplementary figure 1.** The models’ performance for overall survival with p-values of Mann–Whitney–Wilcoxon tests performed for corresponding radiomics-based and radiomics+clinical-based models.


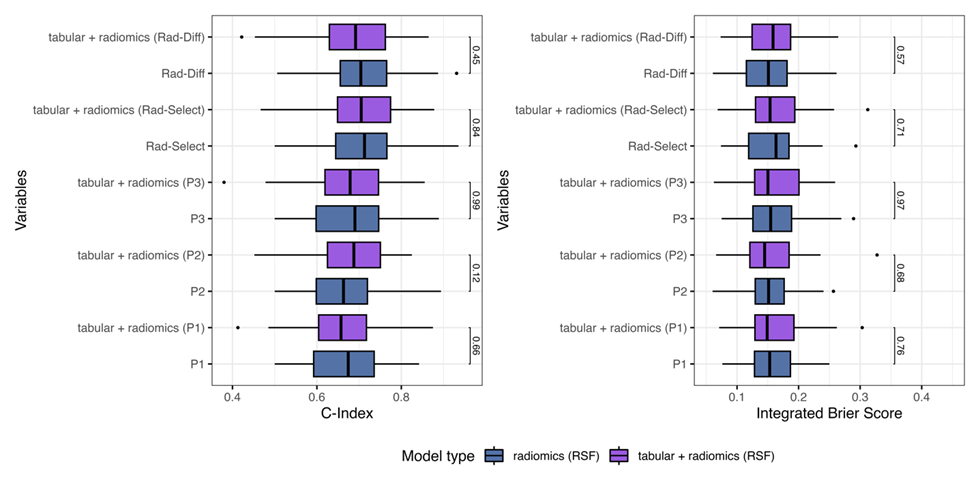


**Supplementary figure 2.** The models’ performance for progression free survival with p-values of Mann–Whitney–Wilcoxon tests performed for corresponding radiomics-based and radiomics+clinical-based models.
